# Preventive and therapeutic effects of rifaximin on hepatic encephalopathy with differential application dosages and strategies: a network meta-analysis

**DOI:** 10.1186/s12876-024-03184-0

**Published:** 2024-03-04

**Authors:** Guihua Fang, Shuna Liu, Bin Liu

**Affiliations:** 1 Department of Infectious Diseases, The Affiliated Hospital of Guangdong Medical University, No.57 Renmin Avenue South, 524000 Xiashan, Zhanjiang, Guangdong China; 2 Laboratory of Hepatobiliary Surgery, The Affiliated Hospital of Guangdong Medical University, 524000 Zhanjiang, Guangdong China

**Keywords:** Rifaximin, Hepatic encephalopathy, Cirrhosis, Systematic review, meta-analysis

## Abstract

**Background:**

Hepatic encephalopathy (HE) is a neuropsychiatric syndrome that affects the prognosis of patients with liver disease and is considered an independent risk factor for hospitalization and death. Rifaximin has been approved for HE treatment. This review will analyze the effect of rifaximin on different stages of HE with differential application dosages and strategies by traditional and network meta-analyses.

**Methods:**

We performed a systematic search of PubMed, EmBase, and Cochrane Library databases up to February 26, 2023, to identify randomized controlled trials (RCTs) about rifaximin for the prevention and treatment of HE. The outcomes included incidence of HE and HE progression, HE reversal, mortality, and adverse effects.

**Results:**

A total of 21 studies were included. In the primary prevention of HE, rifaximin significantly reduced the incidence of HE (OR: 0.66; 95% CI: 0.45, 0.96; *p* = 0.032). In secondary prevention, rifaximin significantly reduced the risk of recurrence in patients who were in remission (OR: 0.38; 95% CI: 0.28, 0.52; *p* < 0.001). In the treatment of minimal HE, rifaximin significantly reduced the breakthrough of MHE to OHE (OR: 0.17; 95% CI: 0.04,0.63; *p* = 0.008). Rifaximin also significantly improved the clinical symptoms of MHE and OHE patients (OR: 3.76; 95% CI: 2.69, 5.25; *p* < 0.001). However, rifaximin did not reduce mortality at any stage in HE patients (OR: 0.79; 95% CI: 0.58, 1.08; *p* = 0.133). Additionally, rifaximin did not increase the risk of adverse effects (OR: 0.96; 95% CI: 0.74, 1.24; *p* = 0.749). In the network meta-analysis, the 400 mg T.I.D. intervention had a relative advantage for HE risks in primary and secondary prevention. In the treatment of MHE, 600 mg b.i.d. was superior in preventing the breakthrough from MHE to OHE.

**Conclusion:**

Rifaximin prevented HE risks and progression and improved clinical symptoms in patients with MHE but did not reduce mortality. For primary and secondary prevention, 400 mg t.i.d. could be considered. 600 mg b.i.d. could be considered in patients with MHE.

**Supplementary Information:**

The online version contains supplementary material available at 10.1186/s12876-024-03184-0.

## Background

Hepatic encephalopathy (HE) is a neuropsychiatric syndrome associated with liver disease, leading to impaired cognitive function, motor activity, and potentially resulting in consciousness and coma [1]. Elevated blood ammonia and inflammation are the main triggers for HE [2]. It is considered one of the most serious complications of decompensated cirrhosis, independently predicting hospitalization and death from liver-related complications [3]. Therefore, prevention and treatment of HE are crucial for patients with liver disease.

The overgrowth and alterations of intestinal bacteria could contribute to hyperammonemia, hyperendotoxemia, and systemic inflammation, leading to the development of HE [4]. Rifaximin is a gastrointestinal selective broad-spectrum antibiotic that is rarely absorbed systemically [5]. It significantly inhibits the proliferation of urease-producing bacteria in the intestine and reduces the production of ammonia and other toxins [6], but it has a low impact on the normal intestinal flora [7]. Plasma ammonia is also considered a predictor of hospitalization and mortality due to liver-related complications in stable cirrhosis outpatients [3].

Additionally, rifaximin is a small intestine-specific pregnane X receptor agonist that inhibits the inflammatory response and reduces the release of proinflammatory factors [8]. Systemic inflammation is very common in decompensated cirrhotic patients and correlates with the severity of HE [9]. This inflammation affects the gut-liver-brain axis, including microglial activation and brain aggregation of proinflammatory factors [10]. Thus, rifaximin may also have a therapeutic effect through systemic anti-inflammatory effects to slow down HE processes.

Rifaximin is an oral nonsystemic antibacterial approved by the FDA for the treatment of patients with HE in 2010 [11]. Rifaximin has also been recommended clinically as an add-on drug for the prevention of HE recurrence [12]. Several traditional and network meta-analyses are available at present and are focused on the treatment of patients with HE. Early published studies suggested that rifaximin has similar efficacy to other active drugs but is better tolerated [13–16]. Subsequent studies have confirmed the benefit of rifaximin in HE treatment and its ability to reduce mortality [17–19] while improving health-related quality of life [20]. However, there is still controversy about its effect on mortality. Harry D Zacharias et al. concluded that, compared to nonabsorbable disaccharides, rifaximin may have no significant impact on mortality, severe adverse events, health-related quality of life, or hepatic encephalopathy. However, when rifaximin is combined with nonabsorbable disaccharides, it may reduce the overall risk of mortality, improve HE, and prevent the occurrence/recurrence of HE [21]. Xianghui Han et al. also suggested that rifaximin did not significantly reduce mortality compared to either a placebo (risk ratio = 0.66 (0.36, 1.20), *p* = 0.176) or other active drugs (risk ratio = 0.99 (0.56, 1.75), *p* = 0.974) [22]. In network meta-analyses, rifaximin showed an effect on reversing minimal HE (MHE) in patients with cirrhosis [23, 24] but was not effective for Overt HE (OHE) patients [25]. The above meta-analyses all ignored the influence of drug dosage and application strategy on the results.

The effective antibacterial concentration of rifaximin has significant implications for its therapeutic efficacy. An in vitro study showed that although rifaximin has a low Minimal inhibitory concentrations (MIC)50 against intestinal ammonia-producing bacteria, the concentration range between the MIC50 and MIC90 is very large (0.004-128 mg/L). When intestinal bacteria are cultured under sub-MIC rifaximin concentrations, they all exhibit a tendency toward drug resistance [26]. In a clinical study, the efficacy of rifaximin in treating small intestine bacterial overgrowth also showed dose-dependent efficacy [27]. Thus, the dose and application strategy of rifaximin will impact the effective drug concentration within the intestinal environment and further influence the inhibitory effect on ammonia-producing bacteria and their drug resistance, which deserves attention.

For HE prevention, the FDA recommended rifaximin 550 mg b.i.d [28].. However, off-label applications have also been commonly adopted. Retrospective studies suggested that the regimen of 550 mg b.i.d. and the off-label regimen of 400 mg t.i.d. had similar effects on alleviating HE. Additionally, a low dose of 400 mg b.i.d. can also similarly reduce serum endotoxin levels after two weeks of treatment compared to a high dose of 600 mg b.i.d [29, 30].. Since rifaximin is expensive, low doses of rifaximin are associated with low treatment costs and can help improve patient compliance and lead to improved treatment efficacy [6, 31]. Therefore, an evidence-based analysis of the differential doses and application strategies of rifaximin in HE and HE-risk patients could help elucidate the discrepancies among previous meta-analyses, help optimize treatment strategies and costs, and improve patient treatment compliance. This review will analyze the effectiveness of different doses and application strategies of rifaximin in patients with HE and with HE risks.

## Methods

This work was performed in accordance with the Preferred Reporting Items for Systematic Reviews and Meta-Analyses (PRISMA) guidelines.

### Search strategy

This review systematically searched literature databases, including PubMed, Embase, and the Cochrane Library, from inception to February 26, 2023. The search terms included “encephalopathy”, “rifaximin”, and “random*”. The Boolean symbols were used to intersect the retrieval results of the above three keywords. The full search formula used in the PubMed database was “(“brain diseases“[MeSH Terms] OR (“brain“[All Fields] AND “diseases“[All Fields]) OR “brain diseases“[All Fields] OR “encephalopathies“[All Fields] OR “encephalopathy“[All Fields]) AND (“rifaximin“[MeSH Terms] OR “rifaximin“[All Fields] OR “rifaximine“[All Fields]) AND “random*“[All Fields]”. Reference lists of published systematic reviews were manually searched to avoid omissions.

### Study selection

Overall, this review planned to include randomized controlled trial (RCT) of rifaximin versus blank control or placebo control or rifaximin dosage-related RCT on patients with HE or HE risk. RCT was excluded if rifaximin was compared with other active agents. Two authors independently performed the literature search and study selection process, and any disagreements were resolved by discussion until a consensus was reached. Two authors first listed their respective viewpoints and support for disagreements. Through the exchange of opinions, most disagreements can typically be resolved. If disagreement persists, the corresponding author will need to participate in the discussion to reach a consensus. If a consensus is still not reached at this point, a voting mechanism (where the minority yields to the majority) based on an odd number of authors is employed to determine the final decision.

The inclusion criteria were as follows: ① the patient had HE or had a risk of HE; ② the intervention group was treated with rifaximin; ③ blank, placebo, or dosage-related control was designed; ④ the study reported one of the outcomes: exacerbation rate (incidence, occurrence, or breakthrough), effective rate (reversal or improvement), or mortality (including patients who received liver transplantation); and ⑤ the study was an RCT design.

Exclusion criteria included the following: ① the control intervention was other (not rifaximin) active therapeutic agents; ② all groups were treated with the same dose of rifaximin as concomitant medication; and ③ duplicated reports.

### Data extraction

A standardized form was developed at the protocol establishment stage. Two authors individually extracted the data according to the predefined form. The extracted contents included the name of the first author, publication year, research location, sample size, type of patients, dose of rifaximin, control intervention, treatment duration and follow-up. The outcomes are listed as follows. The risk of disease progression, including the incidence of HE in primary prevention, the risk of recurrence in secondary prevention, and the risk of exacerbation of MHE to OHE. Disease improvement, including the improvement or reversal of clinical symptoms in HE patients. Number of patients who died or received liver transplantation and the risk of adverse events. After the extraction was complete, the results from the two authors were compared, and any disagreements were resolved by exchanging the viewpoints and voting mechanisms as described above.

### Quality assessment of trials

The quality of trial methodology was carried out independently by two authors, and the tool used was the Cochrane risk of bias tool. The quality of RCT was assessed by seven parameters: randomization sequence generation (the inadequate generation of a randomized sequence increased the risk of bias), allocation concealment (the inadequate concealment of allocations before assignment increased the risk of bias), participant and personnel blinding (the knowledge of the allocated interventions by participants and personnel will be assessed), assessor blinding (the knowledge of the allocated interventions by outcome assessor will be assessed), incomplete outcome data (the item assessed the completeness of participants’ outcome data for each main outcome), selective reporting (the item assessed the selective outcome reporting), and others (the item assessed the potential problems not covered elsewhere) [32].

### Statistical analysis

Traditional meta-analysis was performed first to combine the results. The dichotomous data results were pooled by odds ratios (ORs) with 95% confidence intervals (CIs).

The results of the random-effect model and fixed (common)-effect model were both presented.

The I^2^ statistic was used to assess the heterogeneity among studies. If I^2^ > = 50%, the result based on the random-effect model was adopted; otherwise, the fixed-effect model result was selected. Subgroup analysis was performed by different types of patients or disease phases for disease progression and improvement results. Meta-regression analysis was also performed based on the daily dose of rifaximin and the duration of application. Egger’s and Begg’s tests were used to assess potential publication biases.

A frequentist random effect model-based network meta-analysis (NMA) was then used to compare different rifaximin application strategies. The node splitting method was used to obtain local inconsistencies. The ranking probabilities for each strategy were calculated by the surface under the cumulative ranking curve (SUCRA). The R project (version 4.1.0) with packages “meta (version 6.2-1)” and “netmeta (version 2.8-1)” was used for all analyses.

## Results

**Fig. 1:**
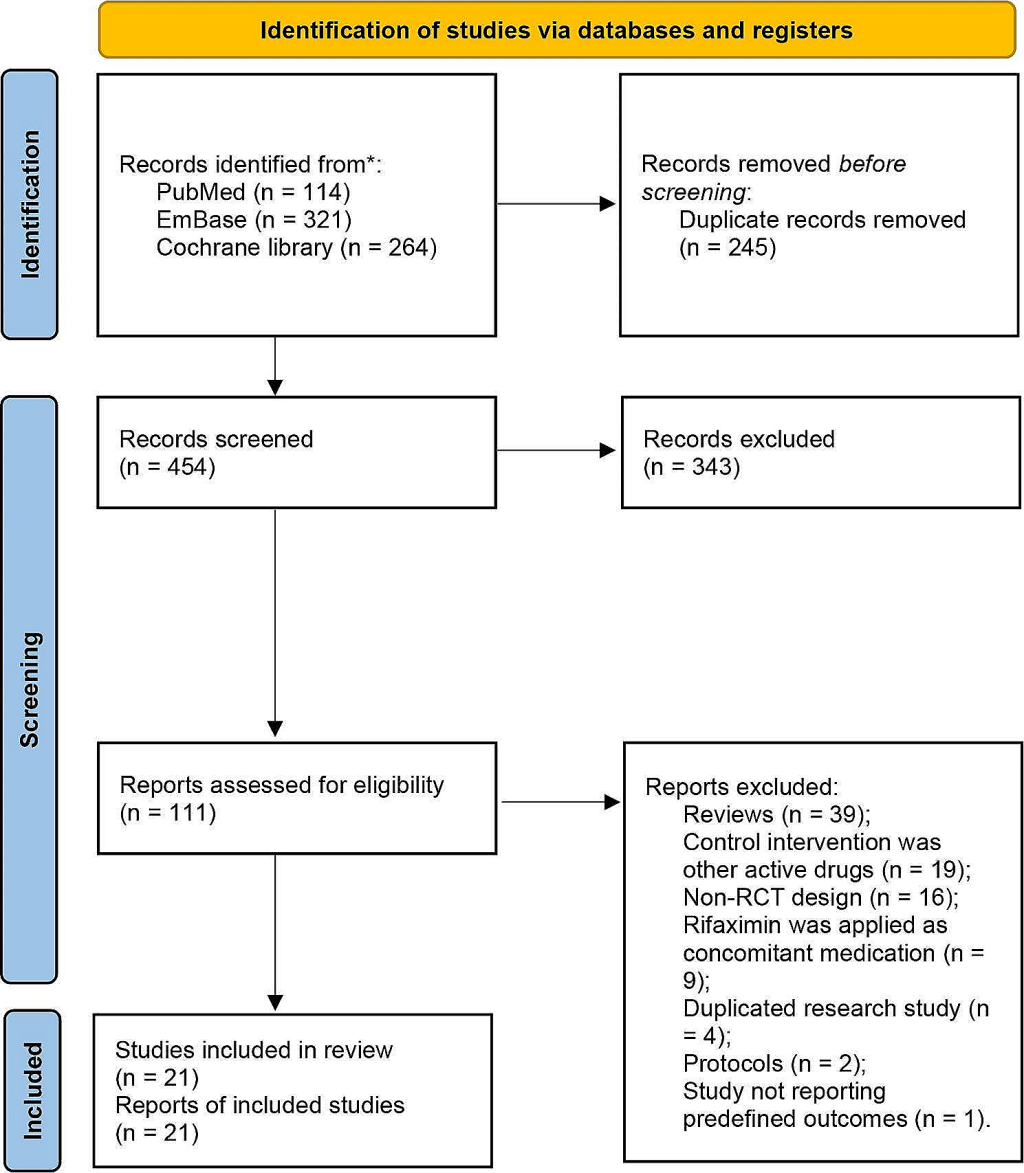
Flow diagram of the literature search and selection process in this analysis

A total of 114 items from PubMed, 321 items from EmBase, and 264 items from the Cochrane Library were harvested. A total of 454 items were obtained after removing duplications. A total of 343 studies were excluded after screening the titles and abstracts. The full texts of the remaining 111 studies were screened, and 90 studies were excluded due to the following reasons: Reviews (*n* = 39); control intervention was other active drugs (*n* = 19); non-RCT design (*n* = 16); rifaximin was applied as concomitant medication (*n* = 9); duplicated research study (*n* = 4); protocols (*n* = 2); study not reporting predefined outcomes (*n* = 1). Finally, 21 articles were included in the analysis [6, 33–53] (Fig. 1; Table 1).

**Table 1:** The characteristics of included studies in this study

Study	Location	Sample size	Patients	Dosage of rifaximin	Control	Duration of treatment#	Follow-up#
Tan W 2023 [6]	China	40	Covert HE	800 mg/1200 mg per day	Black	8 W	8 W
Bajaj JS 2023 [33]	US	71	Cirrhosis	40 mg/80 mg	Placebo	24 W	26 W
Patel VC 2022 [34]	UK	38	Chronic HE	550 mg b.i.d.	Placebo	90D	30D
Abdel Moneim M 2021 [35]	Egypt	100	HE or history of at least one episode	400 mg t.i.d.	Placebo	6 M	6 M
Bureau C 2021 [36]	France	197	Cirrhosis undergoing TIPS	600 mg b.i.d.	Placebo	182D	168D
Zeng X 2021 [37]	China	200	Decompensated cirrhosis	400 mg b.i.d.	Blank	6 M	6 M
Pawar VB 2019 [38]	India	180	Minimal HE	550 mg b.i.d.	Placebo	3 M	3 M
Sarwar S 2019 [39]	Pakistan	75	Decompensated cirrhosis	200 mg b.i.d.; 550 mg b.i.d.	-	6 M	6 M
Hasan S 2018 [40]	India	96	HE	400 mg t.i.d.	Blank	10D	10D
Higuera-de-la-Tijera F 2018 [41]	Mexico	87	Cirrhosis with variceal bleeding	400 mg t.i.d.	Placebo	7d	28D
Butt NI 2018 [42]	Pakistan	130	HE due to decompensated chronic liver disease	550 mg b.i.d.	Blank	10D	10D
Khokhar N 2015 [43]	Pakistan	306	Chronic cirrhosis with a previous episode of HE.	550 mg b.i.d.; 550 mg q.d.	-	6 M	6 M
Sharma K 2014 [44]	India	124	Minimal HE	400 mg t.i.d.	Placebo	2 M	2 M
Ali B 2014 [45]	Pakistan	126	Remission from recurrence HE resulting from cirrhosis	550 mg b.i.d.	Placebo	6 M	6 M
Sharma BC 2013 [46]	India	120	Overt HE	400 mg t.i.d.	Placebo	10D	10D
Sanyal A 2011 [47]	USA	219	Cirrhosis in remission from HE	550 mg b.i.d.	Placebo	6 M	6 M
Sidhu SS 2011 [48]	India	94	Minimal HE	400 mg t.i.d.	Placebo	8 W	8 W
Bajaj JS 2012 [49]	USA	42	Minimal HE	550 mg b.i.d.	Placebo	8 W	8 W
Bass NM 2010 [50]	USA	299	Cirrhosis and HE in remission	550 mg b.i.d.	Placebo	6 M	168D
Riggio O 2005 [51]	Italy	75	Cirrhosis undergoing TIPS	400 mg t.i.d.	Blank	1 M	1 M
Williams R 2000 [52]	UK	54	Mild to moderate HE	600 mg/1200 mg/2400 mg per day	-	7D	7D

Abbreviations: HE: hepatic encephalopathy; TIPS: transjugular intrahepatic portosystemic shunt

#: D: day; W: week; M: month

The included studies were all published after 2000, and the types of patients included patients with cirrhosis with a high risk of HE, patients in HE remission with a history of HE, patients with MHE, and patients with OHE. One study [33] included studies on both patients with cirrhosis and patients with OHE. Regarding the rifaximin dose, one study designed the rifaximin tablet and increased the water solubility, which significantly reduced the applied dose. In the remaining studies, the single daily dose ranged from 550 mg [43] to 2400 mg [52]. The duration of intervention ranged from a minimum of 7 days to 6 months. The follow-up period was similar to the duration of the intervention (Table 1).

**Fig. 2:**
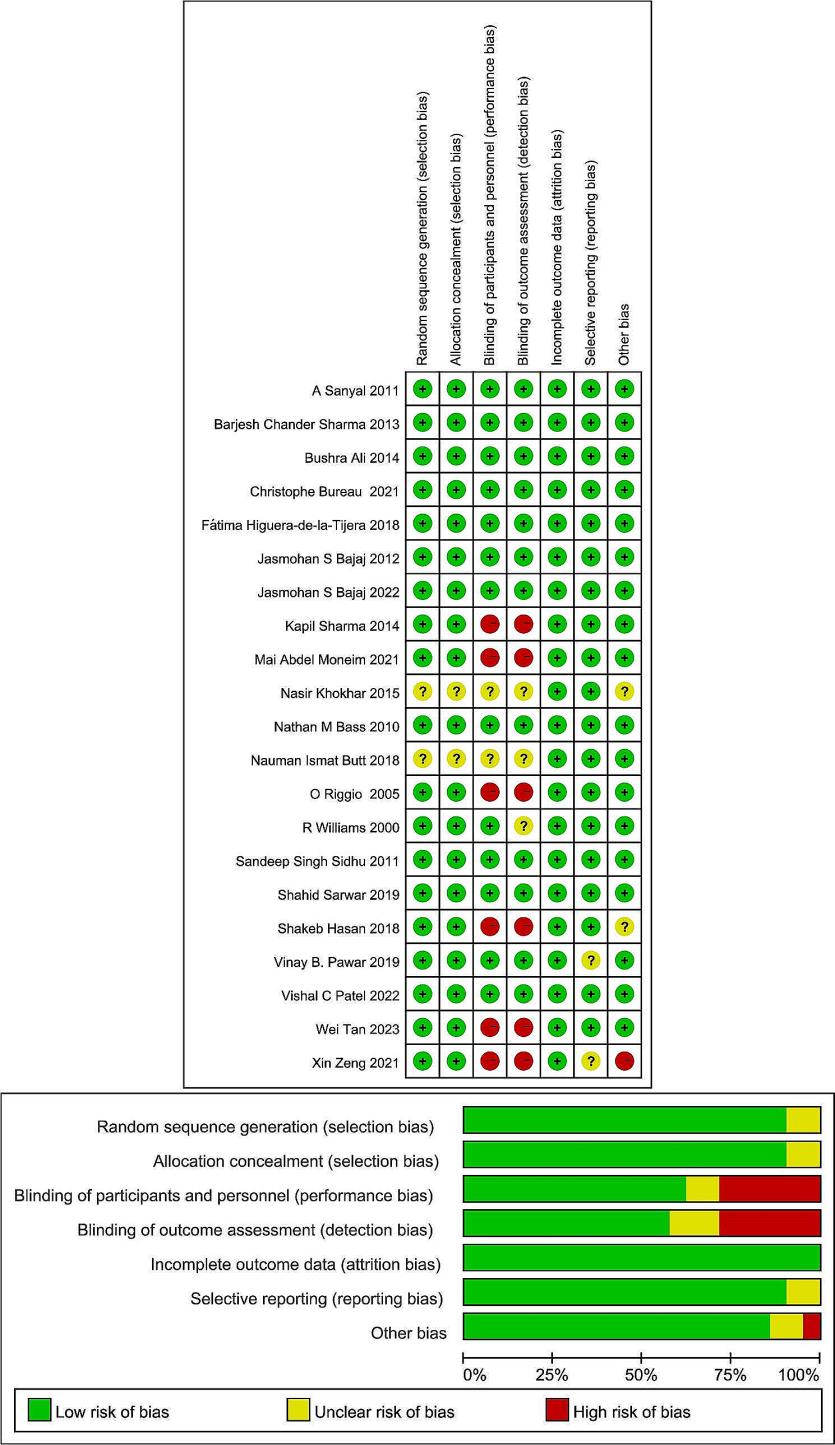
Risk of bias in the included studies

For study design quality, the description of random sequence generation and allocation concealment was unclear in two studies (9.5%), eight studies (38.1%) were not designed for participant and personnel blinding (or were poorly described), and nine studies (42.9%) were not designed for assessor blinding (or were poorly described). Two studies (9.5%) did not report specific values for the results. Other biases included an imbalance of basic characteristics between the intervention and control groups and ambiguity in the description of the results. Overall, more than half of the included studies were well designed, so the overall quality remains satisfactory (Fig. 2).

**Fig. 3:**
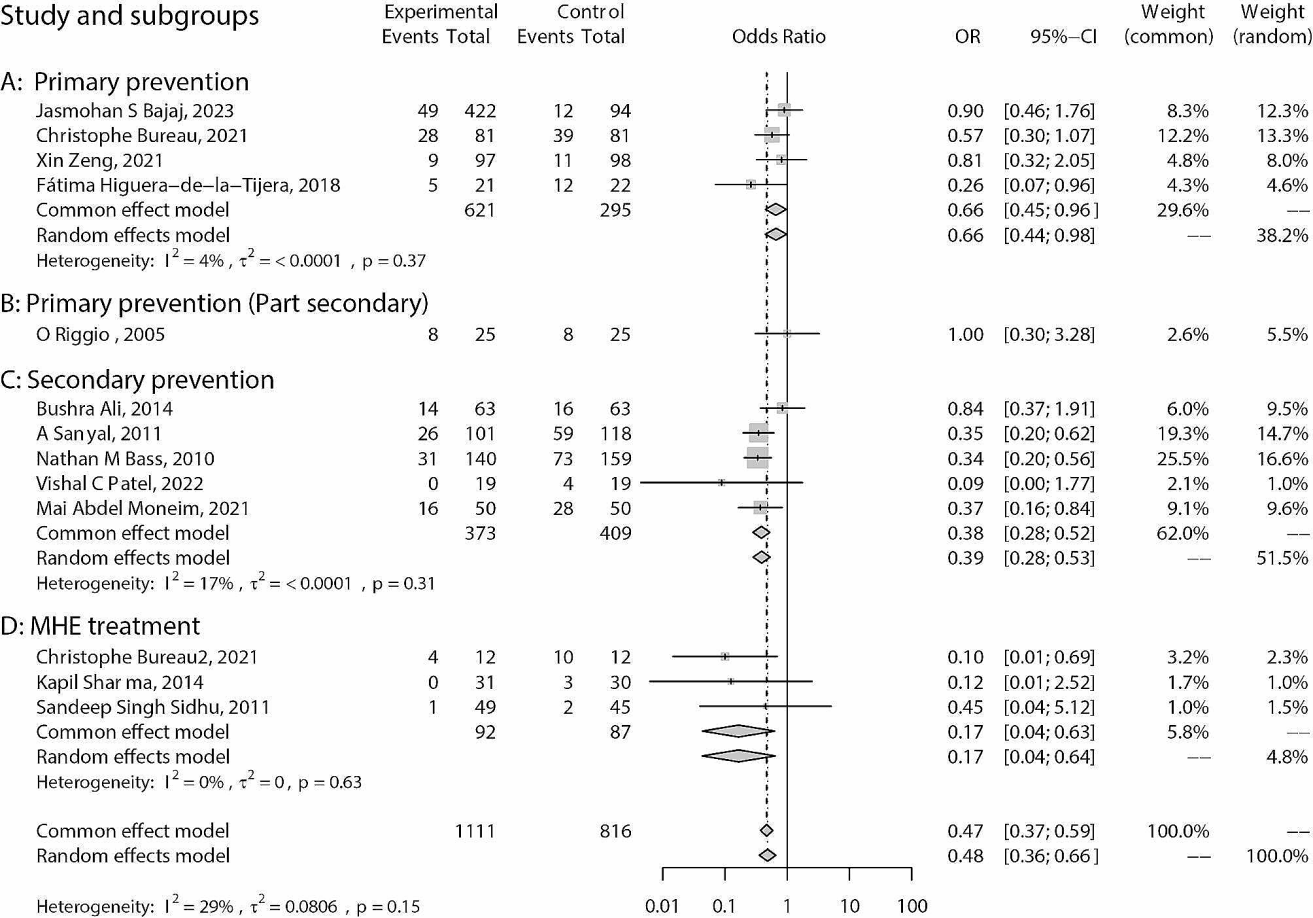
Forest plots of prevention for HE progression between rifaximin and blank/placebo control. **A**: primary prevention of HE; **B**: for patients who undergoing TIPS; **C**: prevention of HE recurrence; **D**: prevention of deterioration from MHE to OHE

For primary prevention of HE, the application of rifaximin significantly reduced the risk of HE compared to the blank/placebo control based on fixed-effect models (OR: 0.66; 95% CI: 0.45, 0.96; *p* = 0.032) (Fig. 3, A). Meta-regression results showed that the daily dose (*p* = 0.383) and duration of application (*p* = 0.180) were not significantly associated with ORs. No potential publication bias was detected (Egger’s test: *p* = 0.425; Begg’s test: *p* = 0.497). Patients who undergo transjugular intrahepatic portosystemic shunt (TIPS) have a higher risk of HE, and in this subgroup analysis, the use of Rifaximin did not significantly reduce the risk of HE occurrence (Fig. 3, B).

For the prevention of HE recurrence, the application of rifaximin could significantly reduce the risk of recurrence according to a fixed-effect model (OR: 0.38; 95% CI: 0.28, 0.52; *p* < 0.001) (Fig. 3, C). Meta-regression results showed that the daily dose (*p* = 0.818) and duration of application (*p* = 0.322) were not significantly associated with ORs. No potential publication bias was found (Egger’s test: *p* = 0.846; Begg’s test: *p* = 0.624).

Rifaximin significantly reduced the risk of deterioration from MHE to OHE (fixed effects model: OR: 0.17; 95% CI: 0.04,0.63; *p* = 0.008) (Fig. 3, D). Due to the small number of related reports, meta-regression and publication bias analyses were not performed.

**Fig. 4:**
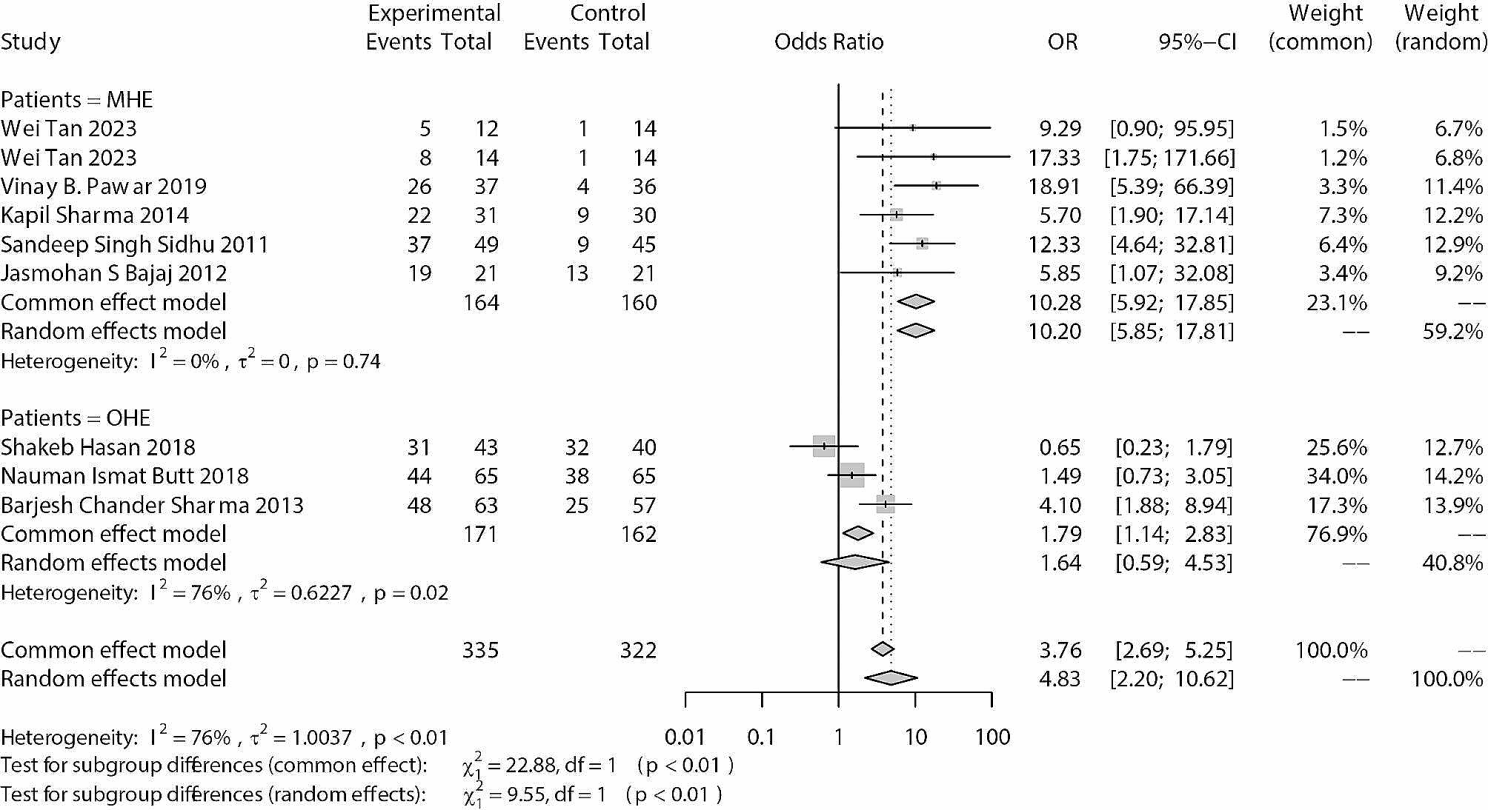
Forest plots of improvement or reversal of MHE between rifaximin and blank/placebo control

For HE improvement or reversal, rifaximin significantly improved the clinical symptoms of HE patients (OR: 4.83; 95% CI: 2.20, 10.62; *p* < 0.001) (Fig. 4). Meta-regression results showed that the daily dose was not associated with ORs (*p* = 0.877), but the duration of application was (β = 0.032, standard error = 0.010, *p* = 0.001). The results may be due to the influence of patient type. In patients with OHE, rifaximin had a limited effect, and the included studies all adopted a 10-day short-term intervention strategy. For MHE patients, the included studies adopted a two- to three-month treatment period. This interpretation was also reflected in the subgroup analysis (Fig. 4). The potential publication bias analysis did not exist in the analysis (Egger’s test: *p* = 0.548; Begg’s test: *p* = 0.297).

Rifaximin did not reduce mortality in patients at any stage (OR: 0.79; 95% CI: 0.58, 1.08; *p* = 0.133) (Additional file 1). Meta-regression results showed that daily dose (*p* = 0.457) and duration of application (*p* = 0.468) were not significantly associated with ORs. No potential publication bias was shown (Egger’s test: *p* = 0.270; Begg’s test: *p* = 0.458). Rifaximin did not increase the risk of adverse effects (OR: 0.96; 95% CI: 0.74, 1.24; *p* = 0.749) (Additional file 2). The results may have potential publication bias (Egger’s test: *p* = 0.029; Begg’s test: *p* = 0.139).

**Fig. 5:**
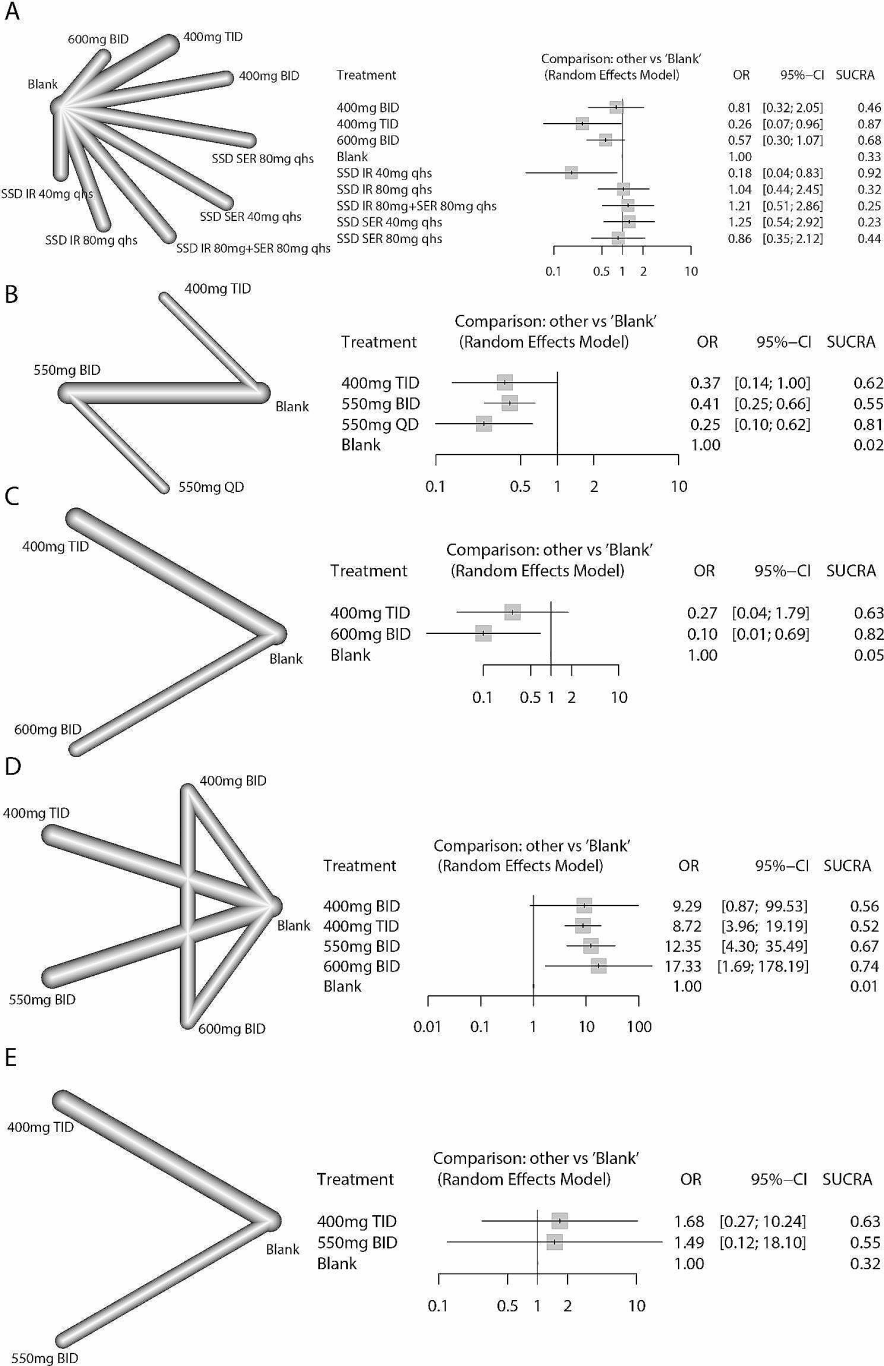
Network plots and network forest plots of patients who received rifaximin compared to blank/placebo controls. **A**: Primary prevention of HE; **B**: prevention of HE recurrence; **C**: prevention of deterioration from MHE to OHE; **D**: MHE reversal; **E**: OHE reversal

In network analysis for HE progression, the strategies included five strategies related to rifaximin increasing the water solubility and six strategies related to classic rifaximin. In primary prevention, no strategy was significantly different compared to the blank/placebo control. Soluble solid dispersion (SSD)- immediate-release (IR) 40 mg rifaximin qhs (SUCRA: 0.92) and 400 mg t.i.d. (SUCRA: 0.87) had relative advantages (Fig. 5, A). In secondary prevention, 550 mg q.d. (SCURA: 0.81) and 400 mg t.i.d. (SUCRA: 0.62) had relative advantages, and all strategies were significantly better than the blank/placebo control (Fig. 5, B). In addition, 600 mg b.i.d. (SUCRA: 0.82) was more effective in preventing the exacerbation from MHE to OHE (Fig. 5, C). For HE improvement, 600 mg b.i.d. rifaximin (SUCRA: 0.743) was also more effective in reversing MHE progression (Fig. 5, D). The 600 mg b.i.d. strategy was lacking in the treatment of OHE, while neither 400 mg t.i.d. nor 550 mg b.i.d. were good at reversing the symptoms of OHE patients (Fig. 5, E).

There is no strategy that can significantly reduce the mortality of patients. SSD rifaximin showed a trend toward reducing the risk of death, but the results were still based on the results of a single study, and more evidence is needed to confirm it (Additional file 3). For adverse effects, rifaximin did not cause additional adverse effects compared to the blank/placebo control. Additionally, 400 mg b.i.d. (SUCRA: 0.866) and 550 mg q.d. (SUCRA: 0.736) had a relatively lower risk of adverse effects (Additional file 4).

## Discussion

HE is one of the most serious complications of decompensated cirrhosis and is considered an independent predictor of hospitalization and death from liver-related complications. Rifaximin was approved by the FDA for HE treatment. The current recommended dose of rifaximin in the treatment of HE is 1200 mg or 1100 mg/day. However, there is still a lack of uniformity regarding the dose and frequency of application [54, 55]. A single study indicated that reducing the administered dose of rifaximin can yield comparable reductions in endotoxin levels to the standard dose [30]. However, additional research is required to determine whether dose reduction maintains therapeutic efficacy for HE and reduces the risk of adverse effects.

Therefore, this work was performed according to the different HE stages, as well as different rifaximin application strategies, by traditional and network meta-analyses. The results showed that rifaximin was able to significantly reduce the risk of HE occurrence in primary prevention, recurrence in secondary prevention, and deterioration from MHE to OHE. Rifaximin also significantly improved the clinical symptoms in patients with MHE but not in patients with OHE. Rifaximin did not benefit mortality at any stage of HE. It also did not add any risk of adverse effects.

In the network meta-analysis, the SSD IR 40 mg and 400 mg t.i.d. strategies had a relative advantage for the prevention of HE risks. 500 mg q.d. and 400 mg t.i.d. had a relative advantage for preventing HE recurrence. Thus, it may be that the 400 mg t.i.d. strategy may be considered in patients without current HE occurrence, while 600 mg b.i.d. may be considered to improve symptoms and prevent deterioration in patients with MHE.

In the results on mortality, this review concluded that the use of rifaximin did not reduce the mortality of patients. In previously published meta-analyses, the combination of rifaximin and lactulose can provide additional benefits in reducing mortality compared with lactulose alone [18, 19]. The conclusion of these two meta-analyses was based on the results of one RCT, as well as conference abstracts or local journal reports. In this review, the majority of included RCTs were unable to confirm that rifaximin could obviously reduce mortality, which is consistent with another recent meta-analysis [22]. It was also concluded that rifaximin was unable to reduce mortality in patients with HE. This review also supplied the effect of rifaximin on mortality in the primary prevention population, and the results still suggested that rifaximin did not significantly reduce mortality in that population.

Due to the original design, this review did not include rifaximin-related RCTs without the “encephalopathy” keyword but reported mortality outcomes. After searching, there was one well-designed RCT relevant to the above topic [56]. The RCT researched the effect of rifaximin on alcohol-related liver disease patients and reported that three patients died in the rifaximin group, but no patient died in the control group. However, the review illustrated that none of the deaths were considered related to rifaximin. It still provided evidence that rifaximin did not provide a potential benefit to the survival outcomes of patients.

The potential targets for HE microbial therapy include regulation of bacterial abundance and microbial products, increasing intestinal barrier function and modification of the host immune response [57]. In the current strategy, prebiotics, probiotics and fecal microbiota transplantation have been used to increase the abundance of beneficial bacteria [1]. At the same time, rifaximin is applied to reduce the abundance of harmful bacteria. Therefore, the characteristics of the intestinal bacteria of patients with HE or a high risk of HE should be first determined. Based on the increase in harmful bacteria or decrease in beneficial bacteria, personalized treatment will be selected for HE treatment.

### Limitations

There are still several limitations in this review. First, this review only included literature published in English, possibly resulting in incomplete data inclusion. Second, this review focused on the HE disease stage and rifaximin drug application strategy. However, the effect of concomitant drugs on HE patients could not be analyzed in this review. Third, this review analyzed the effect of rifaximin compared to a blank/placebo control or dosage-related control. There was no comparative analysis of rifaximin and other active drugs. Fourth, majority of the included RCTs were unable to confirm the reduction of mortality with rifaximin. It was due to these studies did not report mortality outcomes, which may be related to the short follow-up period. For example, in some studies with follow-up periods ranging from 7 days to 1 month, it is very difficult to explore the impact of rifaximin on mortality. In longer-term follow-up studies, rifaximin had no significant effect on reducing mortality, which may be related to the small sample size or its own effectiveness. Therefore, the impact of rifaximin on mortality needs to be further confirmed by the results of well-designed RCTs with large samples size and long-term follow-up.

## Conclusions

In conclusion, Rifaximin prevented HE risks and progression and improved clinical symptoms in patients with MHE but did not reduce mortality at any stage of HE. For primary and secondary prevention of HE, 400 mg t.i.d. strategy may be considered, while 600 mg b.i.d. may be considered to improve symptoms and prevent deterioration in patients with MHE.

### Electronic supplementary material

Below is the link to the electronic supplementary material.

Supplementary Material 1

## Data Availability

The datasets supporting the conclusions of this article are included within the article and its additional files.
