# Supplementary material for: Preventive and therapeutic effects of rifaximin on hepatic encephalopathy with differential application dosages and strategies: a network meta-analysis
Source: BMC Gastroenterol. 2024 Mar 4;24:94. doi: 10.1186/s12876-024-03184-0 (PMC10910798; doi:10.1186/s12876-024-03184-0)

**Additional files**

**Supplementary Figures**

Supplementary Figure 1. Forest plots of mortality of patients who received rifaximin compared to the blank/placebo control.


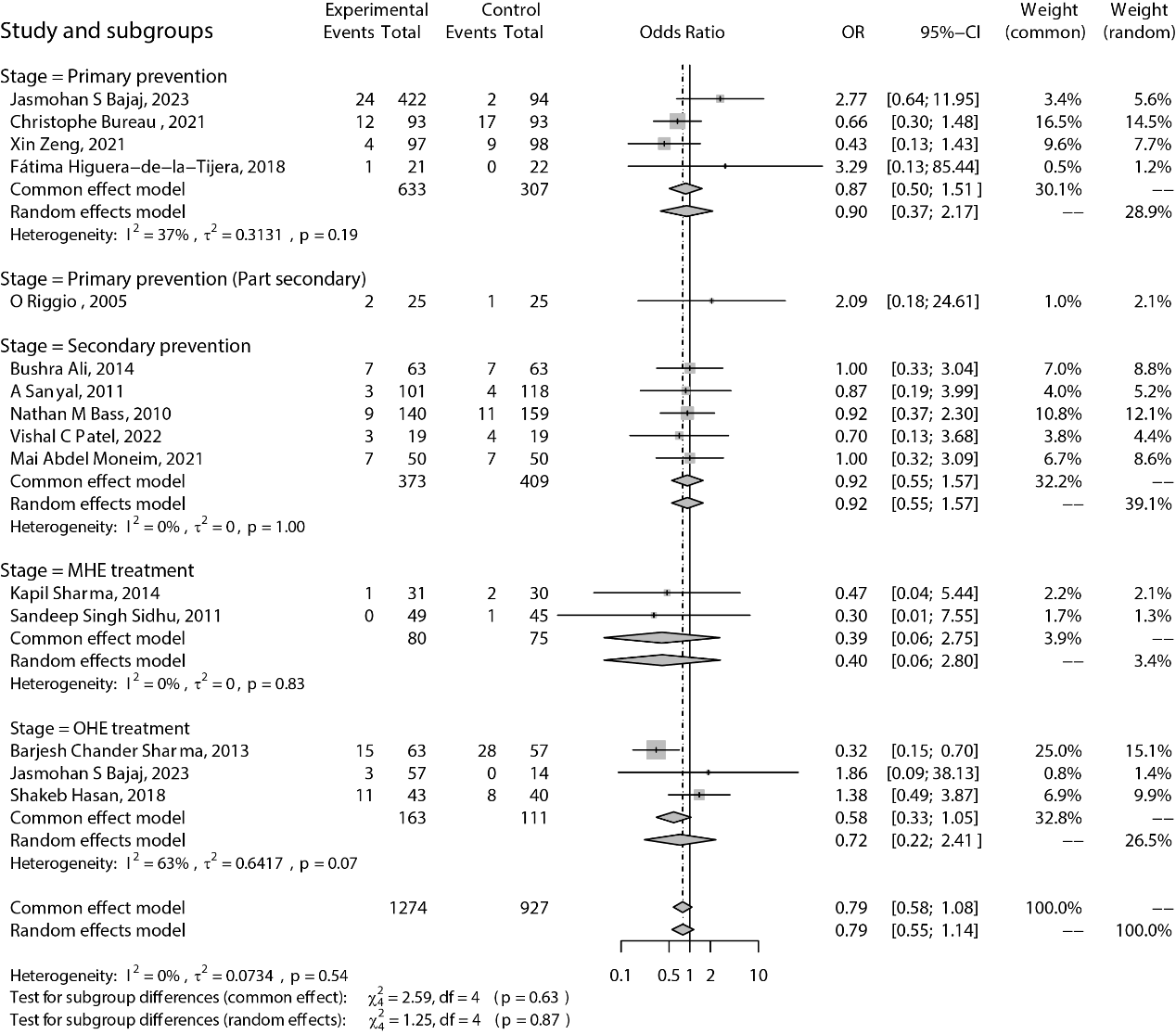


Supplementary Figure 2. Forest plots of adverse effects of patients who received rifaximin compared to the blank/placebo control.


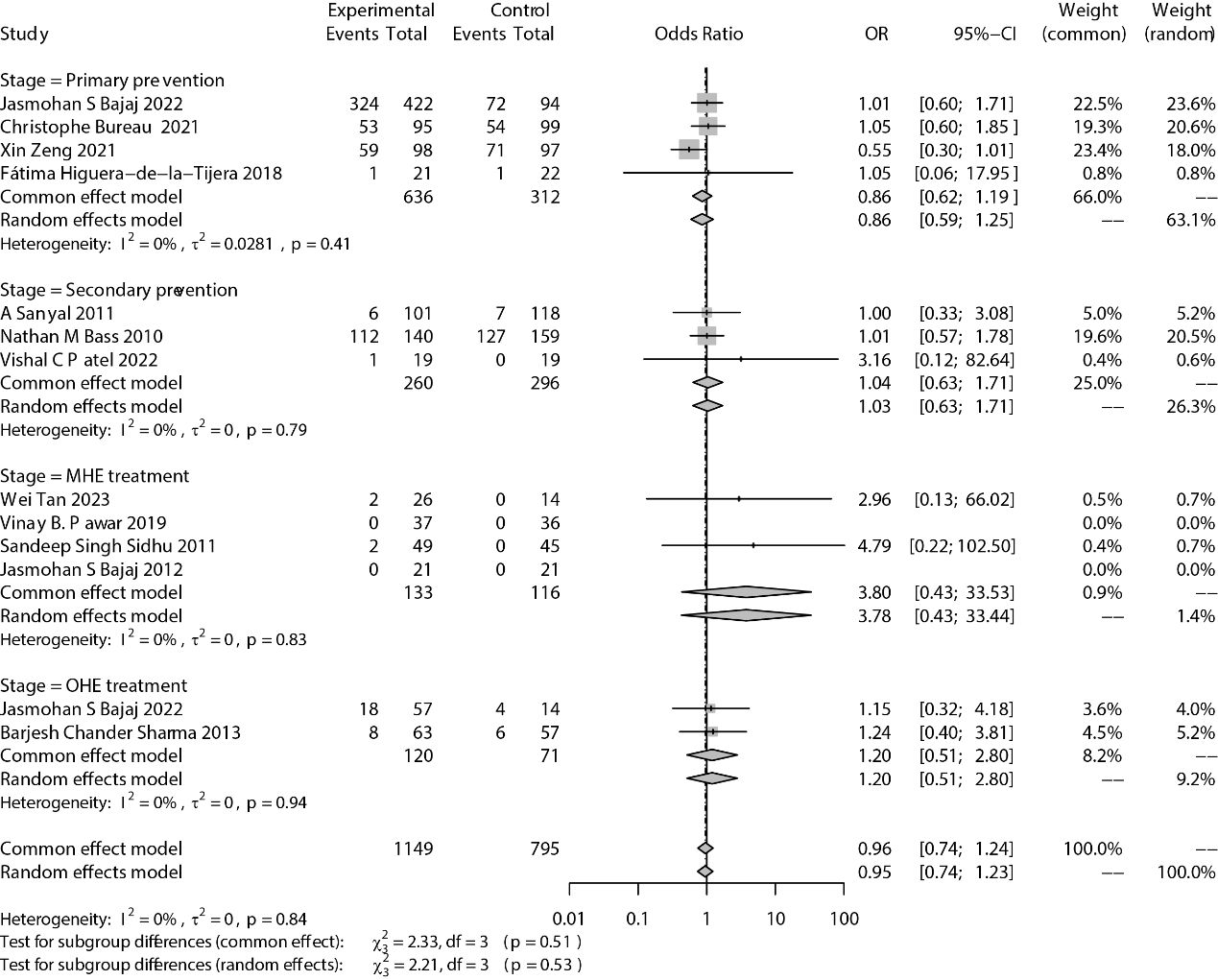


Supplementary Figure 3. Network plots and network forest plots of mortality in patients who received rifaximin compared to the blank/placebo control.


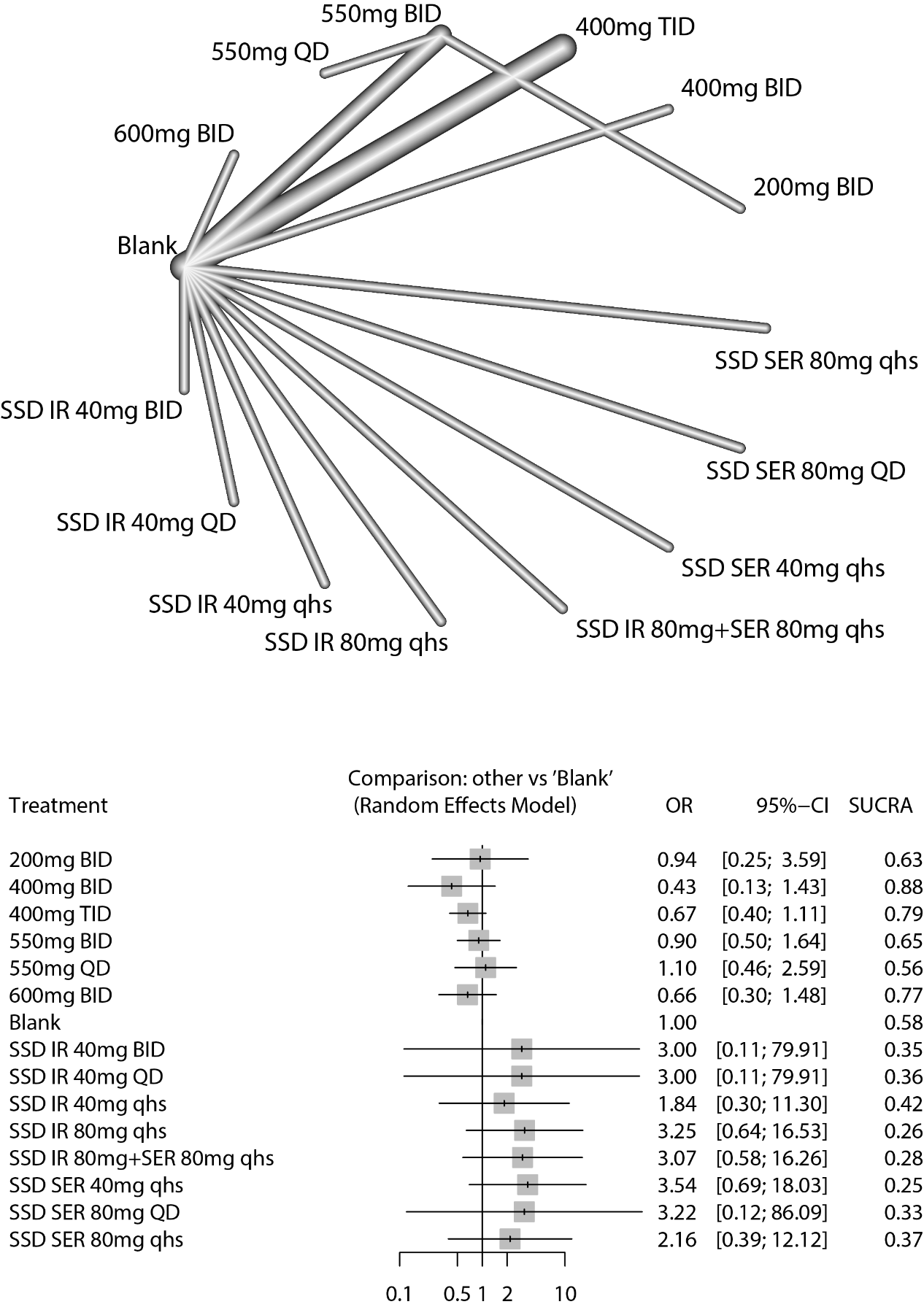


Supplementary Figure 4. Network plots and network forest plots of adverse effects on patients who received rifaximin compared to the blank/placebo control.


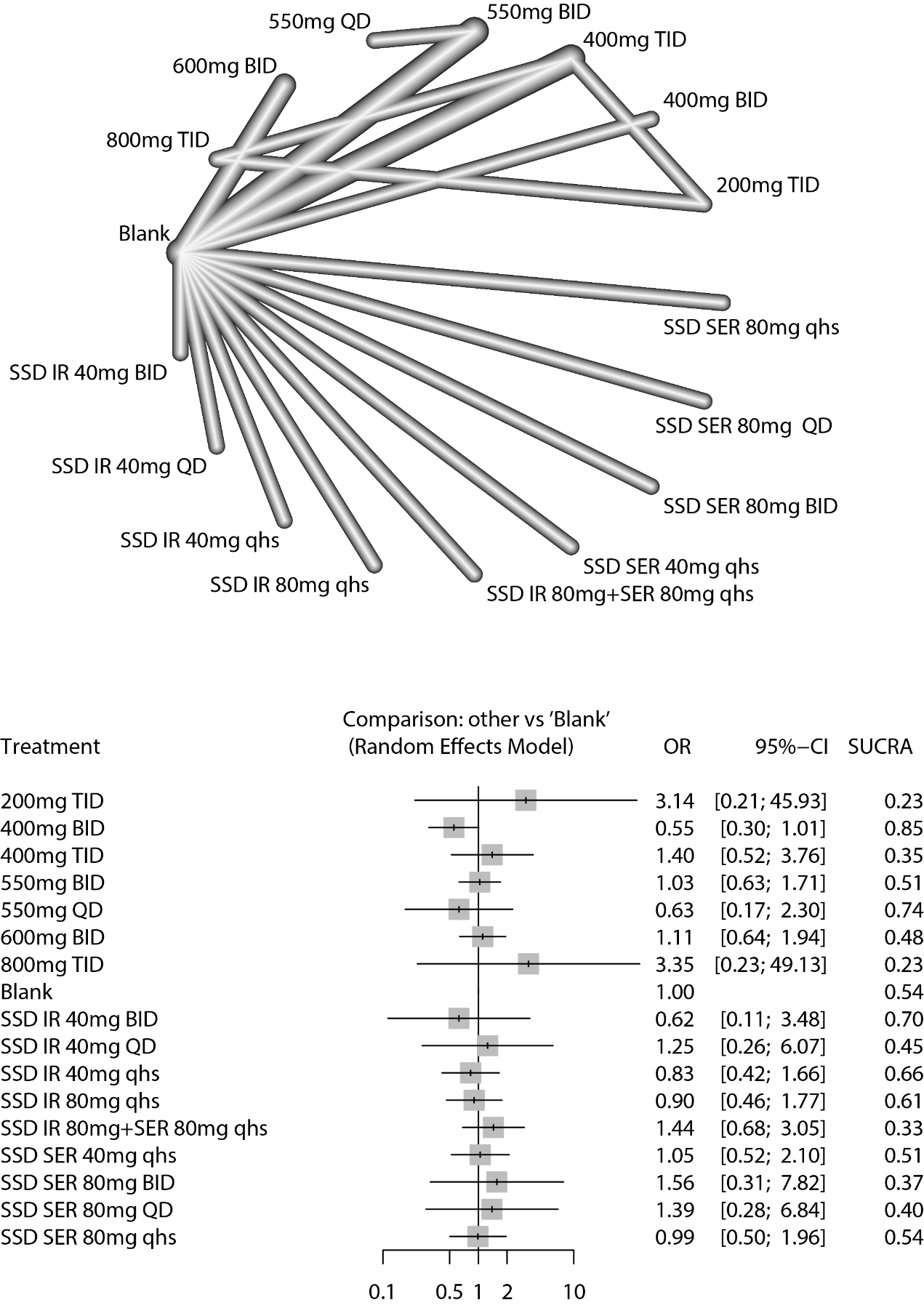

Supplement: Supplementary file 1 — Supplementary Material 1 [file 12876_2024_3184_MOESM1_ESM.docx]
